# Image analysis method for heterogeneity and porosity characterization of biomimetic hydrogels

**DOI:** 10.12688/f1000research.27372.1

**Published:** 2020-12-15

**Authors:** Maryam Jamshidi, Cavus Falamaki

**Affiliations:** 1 Chemical Engineering Department, Amirkabir University of Technology, P.O. Box 15875-4413, Tehran, Iran

**Keywords:** image processing, transformation, noise, filter, pixel, frequency, kernel, distribution, hydrogel, pore

## Abstract

This work presents an image processing procedure for characterization of porosity and heterogeneity of fully hydrated hydrogels based on the analysis of cryogenic scanning electron microscopy (cryo-SEM) images. An algorithm consisting of different filtering, morphological transformation, and thresholding steps to denoise the image whilst emphasizing the hydrogel fibres edges for extracting the pores features is explained. Finally, the information of hydrogel porosity and heterogeneity is presented in form of pore size distribution, spatial contours maps and kernel density dot plots. The obtained results reveal that a non-parametric kernel density plot effectively determines the spatial heterogeneity and porosity of the hydrogel.

## Introduction

Biomimetic engineered hydrogels often serve as 3D microporous extracellular microenvironment mimics in regenerative medicine, tissue engineering ^1^ and *in-vitro* cancer studies ^2^. The physical properties of these gels may provide a mechanical cue to regulate the cell phenotypic activities and functions via cellular mechanotransduction ^3, 4^. Moreover, it has been shown that a change in the stiffness/elasticity of hydrogel is associated with the morphological change in the structure of the hydrogel mesh ^5^. This article presents an image analysis method to characterise hydrogel structure heterogeneity and porosity based on the cryogenic scanning electron microscopy (cryo-SEM) images of fully hydrated hydrogels.

## Methods

### Image processing

The image processing algorithm of the present work has been written as a code in the Python 3.7 language and is able to analyse cryo-SEM images. The present study uses cryo-SEM images of fully hydrated hydrogels adopted with permission from Kaberova *et al.* ^6^ as input data. To detect the pores precisely, the hydrogel fibre edges are highlighted, and a band pass frequency filter is applied to optimize the removal of noise with preservation of the edges of hydrogel walls.

**Figure 1.**
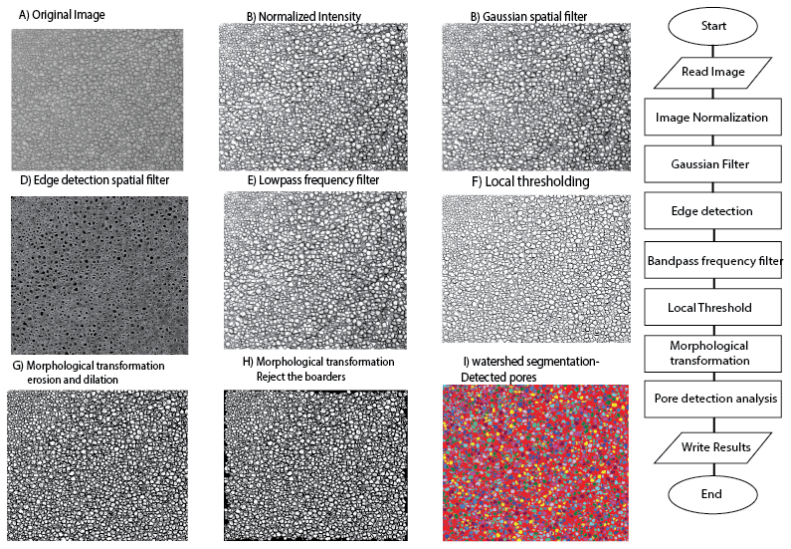
Image processing algorithm of sample cryo-SEM micrograph of glycidyl methacrylate hydrogel crosslinked with 0.3 mol% (ethylene glycol) dimethacrylate. Images on the left show the output images of each step in the processing algorithm and the relevant flowchart is illustrated on the right.

***Pre-processing.*** After loading the image from the specified path, it was first normalized to stretch the Gray level histogram between 0 and 255 and enhance the image contrast. Next, to filter out the noise, a Gaussian (σ =0.7, 3×3) spatial filter convolution was applied to the image. The filtered image is a weighted average of the neighbourhood pixels that better preserve the edges in lower contrast areas while removing the noise ^7^. Afterwards, to extract the edges of the hydrogel fibres, a range of the non-linear 3×3 edge detection filters including Sobel was applied to the image to highlight the locations of sharp intensity transitions. Both the noise and edges belong to the high pass frequencies of the image in essence ^8^ and the edge emphasising filter might introduce some artifacts in these range. Therefore, in the next step, a band pass frequency filter was used to highlight the desired quasi- high range of the frequencies for a better edge detection (refer to Figure 1). 

***Thresholding.*** The binarization of cryo-SEM images of gels is challenging as there is no standard method for thresholding. Since in most of the cryo-SEM images there is uneven illumination, the adaptive threshold technique was undertaken to segment the background and foreground pores. The algorithm calculates the optimal value of threshold based on the weighted mean and standard deviation of the pixel values within the neighbouring window of fixed size for each pixel and outperforms the conventional methods ^9^. In the present work, the adaptive Gaussian threshold has been applied to the image on a window size of 25×25 pixels. The window size should be optimized depending on the density of the details (information) and a lower or greater size may be chosen.

***Morphological transformation.*** The pores touching the boarders of the image were excluded and then basic morphological transformation (erosion×1 and opening×5) was applied to ensure that the isolated pixels both in the background and foreground (pores) were eliminated. For all transformations, a 3×3 elliptical structuring element was applied. Erosion transformation was applied to separate the touching pores and remove the remaining very small pores. Following, the holes within the detected pores were filled up. And lastly, the image was reconstructed based on the erosion and opening results and then a watershed algorithm was used to segment the pores and measure pore properties on the final image. 

To validate the method, the screenshot images of a hydrogel network from a previously published work where the corresponding pore diameters have been reported, were analysed with the proposed method using OTSU threshold and setting 0.05 max watershed threshold. The obtained results of the proposed method (17 µm) were compared with the available reported measurement (15 µm from Figure 4) ^10^.

### Hydrogel porosity and heterogeneity analysis

After performing the pore detection analysis, each single detected pore was associated with the centre of mass and area and the results were exported in a text file. The cryo-SEM image of the sample hydrogel shown in Figure 1A reveals that the hydrogel structure is heterogenous in spatial domain. Therefore, pore size distribution and statistical analysis alone might not represent the spatial heterogeneity and clustering of the detected pores. To quantify and visualize the spatial heterogeneity of the hydrogel, the kernel density estimation function was fitted on the centre of mass of the detected pores on the spatial domain of the cryo-SEM image.

## Results

**Figure 2.**
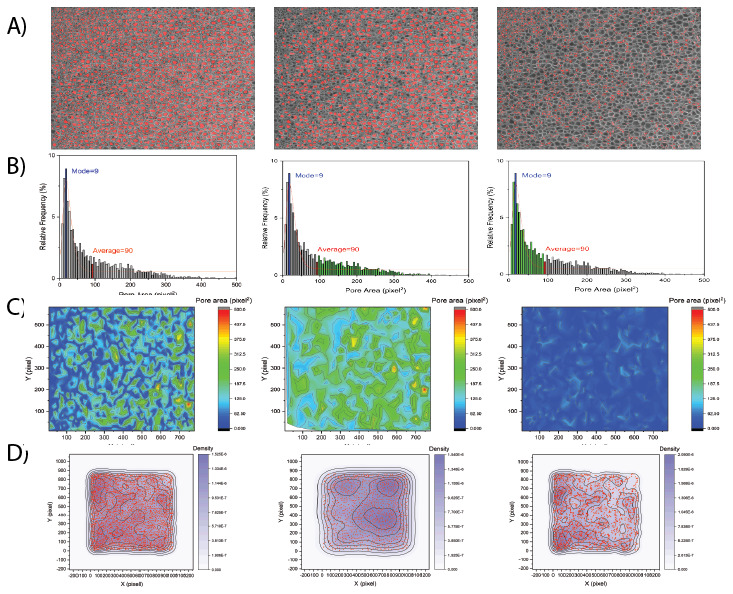
Porosity and heterogeneity analysis of glycidyl methacrylate hydrogel crosslinked with 0.3 mol% di(ethylene glycol) dimethacrylate. **A**) Detected pores of cryo-SEM micrographs. **B**) Pore size distribution. **C**) Spatial contour maps of pore area. **D**) Kernel density estimation dot plots demonstrating the spatial density of detected pores. Data adapted from Kaberova *et al.* ^6^ with permission.

Comparing the obtained results of the proposed method with the available reported measurements of the pore sizes of fluorescent images of hydrogel elsewhere ^10^, the method has been validated with acceptable accuracy. The results of spatial heterogeneity quantification are presented in the form of contour plots ( Figure 2). A higher density value indicates the presence of more pores in the unit of area; therefore, it might represent the location of smaller pore clusters and compactness of the pore clusters. On the other hand, a lower value indicates a less dense area, or an area covered with larger pores. It can be observed from the kernel density contours of a sample image ( Figure 2D) that the distribution of most of the larger pores are almost uniformly dispersed; however, the smaller pores formed clusters in the left-hand side and the corner.

## Conclusion

The algorithm provides an image analysis method for biomaterial science research to investigate the structural heterogeneity of hydrogels. This simple and flexible analysis method allows optimization of different parameters to ideally analyse a broad range of images. We have also demonstrated that based on the data extracted from the image, the kernel density estimation function is a powerful graphical tool to visualize and compare spatial heterogeneity and porosity of the hydrogel. The application of this method can be extended to structural analysis of any fibrous network.

## Data availability

Zenodo: niliou/Hydrogel-pore-size: Hydrogel pore size distribution. https://doi.org/10.5281/zenodo.4308907 ^11^.

This project contains the following underlying data:

- Hydrogel pore size source images (original source image files in TIF format)

Data are available under the terms of the Creative Commons Zero "No rights reserved" data waiver (CC0 1.0 Public domain dedication).

## Code availability

Source code available from: https://github.com/niliou/Hydrogel-pore-size.git

Archived source code at time of publication: https://doi.org/10.5281/zenodo.4308907 ^11^.

License: MIT Licence
