# Supplementary material for: Image analysis method for heterogeneity and porosity characterization of biomimetic hydrogels
Source: F1000Res. 2021 Apr 12;9:1461. Originally published 2020 Dec 15. [Version 2] doi: 10.12688/f1000research.27372.2 (PMC8256190; doi:10.12688/f1000research.27372.2)
Supplement: Peer review history

# Peer review: Reviewer response for version 2

**DOI:** 10.5256/f1000research.55764.r86092

**Published:** 2021-07-02

**Authors:** Susana Rocha

**Affiliations:** 1 Molecular Imaging and Photonics, KU Leuven, Leuven, Belgium

The influence of mechanical and physical cues on cell behaviour has lead to an increasing interest in the quantitative analysis of structural features of hydrogels. The authors here present an algorithm to analyze 2D Cryo-SEM images of a glycidyl methacrylate hydrogel (previous published elsewhere).

 The detailed explanation of the different steps of the algorithm, and the fact that this is open source, are strong points to recommend the indexing of this article. However, there are some points that should be addressed.

1. The pre-processing and thresholding steps are well explained, but there is some information missing in the ‘morphological characterization’ section. The authors mention: ‘And lastly, the image was reconstructed based on the erosion and reopening results and then a watershed algorithm was used to segment the pores and measure pore properties.’ For persons unfamiliar with image processing, a ‘watershed algorithm’ does not say much. Please add some more information, especially on what parameters can be calculated, how are these parameters obtained, and what is the advantage of using a watershed algorithm instead of other analysis (e.g. bubble analysis, as described in the references added in this report).
2. It is not clear how the ‘result’ is calculated. At the end of the section ‘Morphological characterization,’ the authors write: ‘The obtained results of the proposed method (17 µm) were compared….’. It is not clear what the ‘17µm’ refer to. In Figure 2B the average pore equivalent diameter is 12.36µm, so how do the authors obtain the 17 µm value?
3. What do the authors mean with ‘pore equivalent diameter’? This information can be added to the explanation of the watershed algorithm.
4. Can you provide more information on the kernel density estimation function? How does it work and which information can you obtain? It is not clear in the text why you use this function.
5. On figure 1 it is difficult to judge the performance of the different image processing algorithms. Would it be possible to add a magnification? If it is not possible to add it for all the steps, at least a magnified view of the original image and the water-shed segmentation should be added (panels A and I).
6. In figure 2C the axes are in pixels and the pore area is given in µm2. Please change the pixels to µm.
7. General remark: there are no scale bars in the images.

Is the work clearly and accurately presented and does it cite the current literature?

Partly

If applicable, is the statistical analysis and its interpretation appropriate?

Partly

Are all the source data underlying the results available to ensure full reproducibility?

Yes

Is the study design appropriate and is the work technically sound?

Yes

Are the conclusions drawn adequately supported by the results?

Yes

Are sufficient details of methods and analysis provided to allow replication by others?

Yes

Reviewer Expertise:

(fluorescence) microscopy, biomaterials, nanotechnology

I confirm that I have read this submission and believe that I have an appropriate level of expertise to confirm that it is of an acceptable scientific standard.

## References

[1] : Robust pore size analysis of filamentous networks from three-dimensional confocal microscopy. *Biophys J* .2008;95(12) : 10.1529/biophysj.108.135939 6072-80 10.1529/biophysj.108.135939 18835899PMC2599830

[2] : Fast two-dimensional bubble analysis of biopolymer filamentous networks pore size from confocal microscopy thin data stacks. *Biophys J* .2013;104(5) : 10.1016/j.bpj.2013.01.016 1160-9 10.1016/j.bpj.2013.01.016 23473499PMC3870948

[3] : A simplified implementation of the bubble analysis of biopolymer network pores. *Biophys J* .2013;104(12) : 10.1016/j.bpj.2013.05.016 2774-5 10.1016/j.bpj.2013.05.016 23790386PMC3686353

[4] : Response to. *Biophys J* .2013;104(12) : 10.1016/j.bpj.2013.05.015 2776-7 10.1016/j.bpj.2013.05.015 23790387PMC3686351

# Peer review: Reviewer response for version 2

**DOI:** 10.5256/f1000research.55764.r83172

**Published:** 2021-04-20

**Authors:** Miroslava Dušková-Smrčková

**Affiliations:** 1 Institute of Macromolecular Chemistry, Academy of Sciences of the Czech Republic, Prague, Czech Republic

I have reviewed the authors' answers to my points and I liked them and I agree with the modifications/explanations they added. I have no more points to be addressed and recommend the paper for indexing in its current state.

Is the work clearly and accurately presented and does it cite the current literature?

Yes

If applicable, is the statistical analysis and its interpretation appropriate?

Partly

Are all the source data underlying the results available to ensure full reproducibility?

Yes

Is the study design appropriate and is the work technically sound?

Yes

Are the conclusions drawn adequately supported by the results?

Yes

Are sufficient details of methods and analysis provided to allow replication by others?

Yes

Reviewer Expertise:

NA

I confirm that I have read this submission and believe that I have an appropriate level of expertise to confirm that it is of an acceptable scientific standard.

# Peer review: Reviewer response for version 1

**DOI:** 10.5256/f1000research.30250.r79780

**Published:** 2021-03-02

**Authors:** Miroslava Dušková-Smrčková

**Affiliations:** 1 Institute of Macromolecular Chemistry, Academy of Sciences of the Czech Republic, Prague, Czech Republic

It brings a useful piece of computer image analyses software development applicable to quantification of images of heterogeneous materials, namely of 2D projection of pores/distinguishable distributed objects – such as bubbles, particles etc.

 First, a brief generic remark. Presently, a number of image analysis software packages for materials science, commercial as well as open-source, for complex scientific image processing are available. However, it is an increasing challenge for the end user to understand the routine behind the image processing and 2D or 3D structure rendering and sometimes the description of the certain function under “Help” does not offer much possibility to grasp the principle.

 The presented work is particularly concentrated on the analysis of microscopy images of macroporous hydrogels where the advancement of image processing such as the proposed method is still needed: the reason is that we need to investigate relations between hydrogel morphology, microstructure and hydrogel macroscopic properties, mainly mechanical behavior and its effects in biological application – because not everything is known and there are new types of hydrogels of plethora structures being introduced by chemists.

 On top of that, besides the scientific paper containing detailed explanation of itself, Jamshidi and Falamaki made their script (written in Python) available to scientific community – that I found really useful. With more users, we can expect evaluation of high number of samples and possibly building up the software package for more versatile gel characterization. Because of detailed explanation of the hydrogel image processing, the work offers possibility to other interested researchers on the border areas between hydrogel (materials) design and image processing to learn and extend the work. Therefore, I consider publishing this paper with F1000 Research valuable.

 Before indexing the manuscript, I still propose some modification and clarification of few points of discussion – mainly to make the paper speaking to hydrogel designers and chemists who are most of the time users or advanced users but do not have the ambition to be software developers.  

1. A very important point to clarify is the very first sentence of the Abstract. The authors say: “This work presents an image processing procedure for characterization of porosity and heterogeneity of fully hydrated hydrogels based on the analysis of cryogenic scanning electron microscopy (cryo-SEM) images.” Let’s make this clear: analysis of cryo-SEM images may but may not reveal the structure as in hydrated state. When a fully hydrated hydrogel sample is processed for cryogenic EM, it goes through an inevitable freezing step. The morphology of hydrated sample most likely will be altered depending on the conditions of the freezing step but also depending on the particular sample. Freezing, whatever rapid, will cause change in volume and shape of existing pores ^1^, possibly will cause formation of new pores, or will cause some failure, cracks, distortion of the gel phase. Or it will just cause isotropic shrinking of the hydrogel volume. We can’t exactly predict the change. This means that whatever structure of gel the cryo-SEM reveals, it may not correspond to the structure in fully hydrated – meaning swollen in water to equilibrium under given conditions – hydrogel. Only in rather special cases, for example in less swollen and mechanically stronger gels, the gel matrix can resist changes, or in the case of macroscopic gels with arrangement of connected spheres (see Fig. 4 of paper by Kalasova et al in DOI 10.1109/TIM.2020.2995232 ^2^) it can just isotropically contract (as shown in already cited work (https://doi.org/10.3390/polym12030578 ^3^). This all said in the above paragraph does not disrate the impact of the developed image processing – as it can be well applied to images obtained by other methods, too. This point indeed, should be taken into account in the introductory part – pg. 3, where the text again reads: “This article presents an image analysis method to characterise hydrogel structure heterogeneity and porosity based on the cryogenic scanning electron microscopy (cryo-SEM) images of fully hydrated hydrogels.” I propose to modify the formulation similarly as: “This article presents an image analysis method to characterise hydrogel structure heterogeneity and porosity resulting from the treatment of fully hydrated hydrogels – plunge-freezing of hydrated samples done to acquire their cryogenic scanning electron microscopy (cryo-SEM) images.”
2. Several times authors speak about “hydrogel fibers” – I wish to bring up that the form of hydrogel is not fibrous, the gel forms rather thin walls separating the pores in 3D space.
3. There are background and foreground pores mentioned – pls add some schematics what that exactly means and where the reader can see the background vs. the foreground pores on the presented figures.
4. What does the “OTSU” abbreviation stand for and what is OTSU threshold mentioned in part “Morphological transformation”? (abbreviation not explained)
5. The reference where from the Figure 1 was adopted should be given also in the Figure 1 legend.
6. The size of the figures and their resolution even in supplied pptx files is both quite low. Especially the small size of printed letters in the images is critical and must be increased (and improved resolution): legend, axis descriptions, numbers within the Figure 2 are not readable. There are two images both designated Fig.1/ B.
7. The actual results shown on Figure 2 – mean pore size, pore size distribution …is not discussed in numbers. It should also be compared with the original evaluation given in the source article. To determine the size of pores should be the target of the presented method.
8. It would be of interest to discuss the existing limits of the presented method: for example how the method will tackle anisotropic pores, either regularly distributed or clustered or even oriented? – Will the method detect connecting pores, etc? Indeed, this can be the future direction but even then will be worthwhile mentioning.  
9. It would be of interest to adopt the method to evaluate porosity: the void volume fraction in the sample.
10. The thickness of the gel wall separating pores is also of interest and should be also analysed by IA package.  

 Last but not least my comment of Conclusion:

 The material depicted on Figures 1 and 2 should not be called fibrous network – the pores in it look like those in the sponge for washing dishes: pores surrounded by thinner or thicker hydrogel walls of thickness typically 10 ^0^-10 ^1^ um. Indeed, the molecular basis of this material is macromolecular network, that means that all building units molecules are connected to one molecule of a 3D macromolecular network spanning the whole sample space but this molecular nature of the samples indeed is far below resolution of currently available visualization methods.

Is the work clearly and accurately presented and does it cite the current literature?

Yes

If applicable, is the statistical analysis and its interpretation appropriate?

Partly

Are all the source data underlying the results available to ensure full reproducibility?

Yes

Is the study design appropriate and is the work technically sound?

Yes

Are the conclusions drawn adequately supported by the results?

Yes

Are sufficient details of methods and analysis provided to allow replication by others?

Yes

Reviewer Expertise:

macromolecular chemistry

I confirm that I have read this submission and believe that I have an appropriate level of expertise to confirm that it is of an acceptable scientific standard, however I have significant reservations, as outlined above.

## References

[1] : Revealing the True Morphological Structure of Macroporous Soft Hydrogels for Tissue Engineering. *Applied Sciences* .2020;10(19) : 10.3390/app10196672 10.3390/app10196672

[2] : Wide-Cone Angle Phase-Contrast X-Ray Computed Tomography of Synthetic Polymer Materials. *IEEE Transactions on Instrumentation and Measurement* .2020;69(11) : 10.1109/TIM.2020.2995232 8910-8918 10.1109/TIM.2020.2995232

[3] : Microscopic Structure of Swollen Hydrogels by Scanning Electron and Light Microscopies: Artifacts and Reality. *Polymers (Basel)* .2020;12(3) : 10.3390/polym12030578 10.3390/polym12030578 32150859PMC7182949

# Response

**Published:** 2021-03-29

**Authors:** Cavus Falamaki

**Affiliations:** Amirkabir University of Technology, Iran

Author’s Respond to Reviewer:

 Authors would like to sincerely thank reviewer not only for their constructive comments and scientific clarification that improves the scientific quality of the paper, but also for their suggestions that can be used to further develop the current piece of work.

 All modifications made in the manuscript are shown with **.

 Reply to reviewers’ comments are shown with #.

 It brings a useful piece of computer image analyses software development applicable to quantification of images of heterogeneous materials, namely of 2D projection of pores/distinguishable distributed objects – such as bubbles, particles etc.

 First, a brief generic remark. Presently, a number of image analysis software packages for materials science, commercial as well as open-source, for complex scientific image processing are available. However, it is an increasing challenge for the end user to understand the routine behind the image processing and 2D or 3D structure rendering and sometimes the description of the certain function under “Help” does not offer much possibility to grasp the principle.

 The presented work is particularly concentrated on the analysis of microscopy images of macroporous hydrogels where the advancement of image processing such as the proposed method is still needed: the reason is that we need to investigate relations between hydrogel morphology, microstructure and hydrogel macroscopic properties, mainly mechanical behavior and its effects in biological application – because not everything is known and there are new types of hydrogels of plethora structures being introduced by chemists.

 On top of that, besides the scientific paper containing detailed explanation of itself, Jamshidi and Falamaki made their script (written in Python) available to scientific community – that I found really useful. With more users, we can expect evaluation of high number of samples and possibly building up the software package for more versatile gel characterization. Because of detailed explanation of the hydrogel image processing, the work offers possibility to other interested researchers on the border areas between hydrogel (materials) design and image processing to learn and extend the work. Therefore, I consider publishing this paper with F1000 Research valuable.

 Before indexing the manuscript, I still propose some modification and clarification of few points of discussion – mainly to make the paper speaking to hydrogel designers and chemists who are most of the time users or advanced users but do not have the ambition to be software developers.  

- A very important point to clarify is the very first sentence of the Abstract. The authors say: “This work presents an image processing procedure for characterization of porosity and heterogeneity of fully hydrated hydrogels based on the analysis of cryogenic scanning electron microscopy (cryo-SEM) images.” Let’s make this clear: analysis of cryo-SEM images may but may not reveal the structure as in hydrated state. When a fully hydrated hydrogel sample is processed for cryogenic EM, it goes through an inevitable freezing step. The morphology of hydrated sample most likely will be altered depending on the conditions of the freezing step but also depending on the particular sample. Freezing, whatever rapid, will cause change in volume and shape of existing pores 1, possibly will cause formation of new pores, or will cause some failure, cracks, distortion of the gel phase. Or it will just cause isotropic shrinking of the hydrogel volume. We can’t exactly predict the change. This means that whatever structure of gel the cryo-SEM reveals, it may not correspond to the structure in fully hydrated – meaning swollen in water to equilibrium under given conditions – hydrogel. Only in rather special cases, for example in less swollen and mechanically stronger gels, the gel matrix can resist changes, or in the case of macroscopic gels with arrangement of connected spheres (see Fig. 4 of paper by Kalasova et al in DOI 10.1109/TIM.2020.2995232 2) it can just isotropically contract (as shown in already cited work (https://doi.org/10.3390/polym12030578 3). This all said in the above paragraph does not disrate the impact of the developed image processing – as it can be well applied to images obtained by other methods, too. This point indeed, should be taken into account in the introductory part – pg. 3, where the text again reads: “This article presents an image analysis method to characterise hydrogel structure heterogeneity and porosity based on the cryogenic scanning electron microscopy (cryo-SEM) images of fully hydrated hydrogels.” I propose to modify the formulation similarly as: “This article presents an image analysis method to characterise hydrogel structure heterogeneity and porosity resulting from the treatment of fully hydrated hydrogels – plunge-freezing of hydrated samples done to acquire their cryogenic scanning electron microscopy (cryo-SEM) images.”

# The authors agree and admit that in the paper it should be clarified that there are some artifacts associated with the imaging methods specially cryo-SEM and proper referencing should be made.

 # The abstract is revised accordingly, and some clarifications is added to the introduction part to cover the reviewer’s points:

 **This work presents an image processing procedure for characterization of porosity and heterogeneity of hydrogels network mainly based on the analysis of cryogenic scanning electron microscopy (cryo-SEM) images and can be extended to any other type of microscopy images of hydrogel porous network.

 #The following paragraph is modified in the introduction part:

 **This article presents an image analysis method applied to characterise hydrogel structure heterogeneity and porosity resulting from the treatment of fully hydrated hydrogels during plunge-freezing to acquire their cryogenic scanning electron microscopy (cryo-SEM) images. It should be mentioned that during the process of sample preparation for cryo-SEM microscopy, the sample undergoes a probable morphological alteration, furthermore the process itself might introduce artifacts according to the nature of the material and swelling rate. Readers are encouraged to refer to the studies specialized on the imaging of different hydrogel network by Kalasova et. al ^1^ and Pradny et. al ^2^. 

  

- Several times authors speak about “hydrogel fibers” – I wish to bring up that the form of hydrogel is not fibrous, the gel forms rather thin walls separating the pores in 3D space.

# Authors acknowledge the point, in the manuscript wherever the term “fibre” is used, replaced with “hydrogel walls”.

  

- There are background and foreground pores mentioned – pls add some schematics what that exactly means and where the reader can see the background vs. the foreground pores on the presented figures.

# Authors agree that there is a need to clarify the terms used to explain the method. The image of the hydrogel is first segmented into two categories of the hydrogel walls and pores. Because in this analysis we aim to detect the pores, therefore the pores segment is foreground (value 1 – white), and the walls are considered as background (value 0- black). Accordingly, the figure 1 has been edited and a small schematic showing foreground and background is added to the figure 1.

 # Following section has been corrected also for more clarification in “Thresholding” section and also a schematic is added to Figure 1:

 **Since in most of the cryo-SEM images there is uneven illumination, the adaptive threshold technique was undertaken to segment the background (hydrogel walls, value=0 black) and foreground pores (pores, value=1 white).

 **Figure 1. Image processing algorithm of sample cryo-SEM micrograph of glycidyl methacrylate hydrogel crosslinked with 0.3 mol% (ethylene glycol) dimethacrylate. Images on the left show the output images of each step in the processing algorithm and the relevant flowchart is illustrated on the right to distinguish foreground (pores) from background (hydrogel walls). The source image adapted from Kaberova *et al.* ^3^ with permission.

- What does the “OTSU” abbreviation stand for and what is OTSU threshold mentioned in part “Morphological transformation”? (abbreviation not explained)

# There was a typo here, Otsu’s method is one of the methods used for image binarization or thresholding. The following sentence in under “Morphological transformation” is edited:

 **To validate the method, the screenshot images of a hydrogel network from a previously published work where the corresponding pore diameters have been reported, were analysed with the proposed method using Otsu’s thresholding method and setting 0.05 max watershed threshold.

 # in the corresponding python script under thresholding section, both methods of binarization (Otsu’s and Adaptive are provided), user can comment each line and use what is best to process their image. We have used Otsu’s for validation, and adaptive method is suggested for cryo-EM images.

  

- The reference where from the Figure 1 was adopted should be given also in the Figure 1 legend.

# The comment has been considered and the legend for figure 1 is revised as bellow:

 *Figure 1. Image processing algorithm of sample cryo-SEM micrograph of glycidyl methacrylate hydrogel crosslinked with 0.3 mol% (ethylene glycol) dimethacrylate. Images on the left show the output images of each step in the processing algorithm and the relevant flowchart is illustrated on the right to distinguish foreground (pores) from background (hydrogel walls). The source image adapted from Kaberova *et al.* ^3^ with permission.

- The size of the figures and their resolution even in supplied pptx files is both quite low. Especially the small size of printed letters in the images is critical and must be increased (and improved resolution): legend, axis descriptions, numbers within the Figure 2 are not readable. There are two images both designated Fig.1/ B.

#The comment considered, the resolution of the figures increased and high-quality images in actual sizes has been provided in power point files as attachment.

  

- The actual results shown on Figure 2 – mean pore size, pore size distribution …is not discussed in numbers. It should also be compared with the original evaluation given in the source article. To determine the size of pores should be the target of the presented method.

# Authors consider the comment; however, it should be noted that for this paper we have analysed only one image (Figure 1. e) of the source article by Kaberova et. al 3. This image is related to sample G0/0.3 in the source article which the pore size is reported between 2-40 µm (we assume that these values are based on equivalent diameter) for this sample according to Table 1. Using the current method, the average equivalent diameter of the detected pores is equal to 9.45 pixels or 12.36 µm (pixel to microns scale of the image = 500 µm/ 382 pixel). The actual pore size distribution is updated in Figure 2, from this image it is apparent that the measured pore size distribution by this method sit between 2 – 36 µm.

 # the correction applied to the Results section of the manuscript:

 * Moreover, the average equivalent diameter of the pores of the analysed source image (adopted from Kaberova et. al ^3^) was equal to 12.36 µm with the corresponding pore size distribution range of 2-36 µm. The comparison of the results with the range reported by Kaberova et al. ^3^ (2–40 µm) shows a good agreement and reliability of the presented method as it is shown in Figure 2B.

- It would be of interest to discuss the existing limits of the presented method: for example how the method will tackle anisotropic pores, either regularly distributed or clustered or even oriented? – Will the method detect connecting pores, etc? Indeed, this can be the future direction but even then will be worthwhile mentioning.

# This is a very interesting point picked up by reviewer. Authors clarify that the current method does not classify the hydrogel network as “clustered” or “distributed” and we have not done any statistical analysis to focus on quantification of anisotropic properties of the hydrogel network. However, structural and cluster analysis of the gel network is the topic of the next paper currently developing by one of the team members. The result and relevant script of the next work will be available to scientific community after publishing.

 # For the detection of the connecting pores, we have applied watershed transform which is a well-established method to separate touching objects, however authors admit that there are more advanced and complex methods/ pipelines to segment the pores or hydrogel walls. The might be of interest to apply a recently developed methods that combines the classic “watershed” segmentation with “deep learning” ^4^ to increase the level of accuracy and performance of the segmentation method of the analysis package.    

 # The following sentences under Conclusion part is revised in the manuscript:

 *The application of this method can be extended to structural analysis of any other porous network. Furthermore, it is worth mentioning that applying more sophisticated segmentation methods ^4^ to combine classical transformation with deep learning models in the future works might improve the accuracy and performance of the method to distinguish touching pores and separate hydrogel walls.   It would be of interest to adopt the method to evaluate porosity: the void volume fraction in the sample.

 # Authors acknowledge the reviewer’s point. The void fraction calculation has appended to the script and it is printed in the console when the script run has ended (open images should be closed). We consider 2D void fraction = total areal of pores / image area.

 # Script new version (R2 is uploaded on github accordingly).

https://github.com/niliou/Hydrogel-pore-size/blob/main/Hydrogel%20Pore%20Size%20Distribution%20-%20R2%20Final.py

  

- The thickness of the gel wall separating pores is also of interest and should be also analysed by IA package.  

# We admit the comment, the present method can also be used to measure wall thickness of the gel. The only change to be made is the background and fore ground that should be reversed (fore ground is of interest = white) instead of pores we need to bring walls to the front or white. Another point is that for thresholding some values should be tweaked that is commented in the corresponding section in the script.  Therefore, by inversing the image look up table the wall thickness can be measured. For the source image we have:

 # should be noted that in the results file the minimum axis value should be considered as closet parameter describing hydrogel walls. The value of the average wall thickness for the source image is equal to 4.79 pixels or 6.26 µm.

           # The following clarification has been made to the Image processing section:

 *To detect and measure the thickness of the hydrogel walls, the look up table of the binary image should be inversed to bring hydrogel walls in foreground (white).

 #The relevant hints are also commented in the script.

 #To invert the look-up table of the image in the first part, Then, line 130 should be uncommented and used for hydrogel wall thickness measurement,

 the input image in the command line (130) could be any of the filtered images that we have included in the script, so the end user can choose the best filter that gives the best results, in the above we have chosen “DoGNN” image which is filtered image by Difference of Gaussian method. In the script we have several filters (lines 45-84) + bandpass filter lines (103-104) that mainly focuses on edge feature detection. We have commented in the script describing what filters are applied in each section. So, user can choose which one of these filters can extract the wall features the best. Line 144 also can be commented in case “hydrogel wall thickness” is being measured.

 Last but not least my comment of Conclusion:

 The material depicted on Figures 1 and 2 should not be called fibrous network – the pores in it look like those in the sponge for washing dishes: pores surrounded by thinner or thicker hydrogel walls of thickness typically 100-101 um. Indeed, the molecular basis of this material is macromolecular network, that means that all building units molecules are connected to one molecule of a 3D macromolecular network spanning the whole sample space but this molecular nature of the samples indeed is far below resolution of currently available visualization methods.

 # Authors appreciate reviewer’s attention and their explanation to improve the scientific value of the work as well as authors understanding of the nature of hydrogel network. Anywhere in the manuscript the term “fibrous” is revised and replaced.

 1.            Kalasová D, Břínek A, Šlouf M, Dušková-Smrčková M, Zikmund T, Patáková Z, et al. Wide-Cone Angle Phase-Contrast X-Ray Computed Tomography of Synthetic Polymer Materials. IEEE Transactions on Instrumentation and Measurement. 2020; **69**(11):8910-8.

 2.            Přádný M, Dušková-Smrčková M, Dušek K, Janoušková O, Sadakbayeva Z, Šlouf M, et al. Macroporous 2-hydroxyethyl methacrylate hydrogels of dual porosity for cell cultivation: morphology, swelling, permeability, and mechanical behavior. 2014; **21**(11):1-12.

 3.            Kaberova Z, Karpushkin E, Nevoralová M, Vetrík M, Šlouf M, Dušková-Smrčková M. Microscopic Structure of Swollen Hydrogels by Scanning Electron and Light Microscopies: Artifacts and Reality. Polymers. 2020; **12**(3):578.

 4.            Bai M, Urtasun R, editors. Deep watershed transform for instance segmentation. 2017.
